# Radiotherapy combined with anti-PD-1 immunotherapy promotes ferroptosis-driven control of hepatocellular carcinoma

**DOI:** 10.1038/s41435-025-00370-2

**Published:** 2025-12-19

**Authors:** Ting Dou, Xianggao Zhu, Hong Li, Runmei Wang, Jiepu He, Hongwei Geng, Wei Zhang, Qin Yu, Haiping Zhao, Hao Yang

**Affiliations:** 1 Department of Radiation Oncology, Peking University Cancer Hospital (Inner Mongolia Campus) & Affiliated Cancer Hospital of Inner Mongolia Medical University, Huhhot, Inner Mongolia Autonomous Region China; 2 Inner Mongolia Medical University, Huhhot, Inner Mongolia Autonomous Region China; 3 Key laboratory of Carcinogenesis and Translational Research (Ministry of Education/Beijing), Department of Radiation Oncology, Peking University Cancer Hospital & Institute, Beijing, China; 4 Department of Abdominal Tumor Surgery, Affiliated Hospital of Inner Mongolia Medical University, Huhhot, Inner Mongolia Autonomous Region China

**Keywords:** Genetics, Cancer genetics

## Abstract

Combination of radiotherapy (RT) and anti-PD-1 immunotherapy (IO) has shown significant efficacy in treating hepatocellular carcinoma (HCC). Nevertheless, yet the underlying mechanisms remain incompletely understood. A Hepa1-6 mouse HCC model was established to explore the anti-tumor mechanism of combination therapy in HCC. Notably, combination therapy effectively inhibited tumor growth in mice bearing Hepa1-6 tumors. Through MeRIP-sequencing, we indicated that combination therapy increased m6A modification and reduced mRNA expression of Hspb1, a negative regulator of ferroptosis, in tumors from mice. Both combination therapy and Hspb1 downregulation significantly induced Hepa1-6 cell ferroptosis. Metabolomics analysis revealed that Hspb1 downregulation further promoted abnormal lipid metabolism in Hepa1-6 tumor-bearing mice, enhancing pro-ferroptosis effects of combination therapy. Meanwhile, Hspb1 downregulation further enhanced RT and IO-induced anti-tumor immune response in tumor-bearing mice, as evidenced by significantly elevated numbers of cytotoxic CD8 + T cells. Additionally, combination therapy also significantly downregulated RNA demethylase Alkbh5 in tumor-bearing mice. Overexpression of Alkbh5 increased Hspb1 expression and inhibited ferroptosis, indicating that Alkbh5 regulates ferroptosis through targeting Hspb1. Targeting Alkbh5/Hspb1/ferroptosis axis may enhance anti-tumor effects in combination therapy, highlighting a potential therapeutic approach for HCC.

## Introduction

Hepatocellular carcinoma (HCC) is a leading cause of cancer-related death worldwide, which is characterized by high incidence rate and high mortality [1, 2]. In recent decades, despite considerable progress in cancer therapy, the prognosis for patients with HCC remains poor [3–5]. Uncovering effective methods for HCC treatment are urgently. Recently, radiotherapy (RT) and immunotherapy (IO) have merged as promising treatment modalities for malignant tumors [6]. RT, as a nonsurgical local treatment method, has been shown to be effective in preventing early-stage or advanced HCC [7, 8]. RT can eliminate primary and metastatic tumors via high doses of radiation [9]. IO is another promising therapeutic approach for advanced HCC [10]. IO can manipulate the body’s own immune system to identify and eliminate cancer cells [11]. Evidence have shown that programmed death ligand 1 (PD-L1)/(programmed death 1) PD-1 blockade IO has been approved for treating various cancers including HCC [12, 13]. Combination of RT and IO exhibited great promises in solid tumors [14]. Theelen et al. reported that compared to IO (pembrolizumab, an anti-PD-1 agent) alone, pembrolizumab combined with radiotherapy treatment greatly elevated the median progression-free survival and median overall survival in patients with metastatic non-small-cell lung cancer (NSCLC) [14]. Kin et al. showed that compared to anti-PD-L1 or radiation treatment group, combination of anti-PD-L1 and radiation obviously extended the survival in a murine HCC model [15]. Li et al. reported that RT combined with lenvatinib plus PD-1 inhibitor had a great therapeutic effect in HCC patients with portal vein tumor thrombus [16]. Nevertheless, the potential anti-tumor mechanism of anti-PD-1 combined with RT in HCC remains largely unstudied.

Ferroptosis is a novel type of cell death that is triggered by lipid peroxidation [17]. Ferroptosis plays a pivotal role in cancer development [18]. Induction of ferroptosis could inhibit the progression of HCC [19]. In this study, we screened the differentially m6A-modified genes between control and anti-PD-1 plus RT (combination treatment) groups by using MeRIP-seq. The analysis revealed that compared to control group, m6A modification of heat-shock protein B (Hspb1) mRNA level was elevated, but Hspb1 mRNA level was notably reduced in mice bearing Hepa1-6 tumors in the combination treatment group. Evidence has shown that HSPB1 is a negative regulator of ferroptosis [20], and its knockdown has been shown to enhance erastin-induced ferroptosis in cancer cells [20]. We further observed that combination treatment notably decreased the expression of alkB homolog 5 (Alkbh5), a RNA demethylase [21]. Overexpression of Alkbh5 elevated Hspb1 expression and suppressed ferroptosis in Hepa1-6 cells, suggesting that Alkbh5 modulates ferroptosis by targeting Hspb1. It has been shown that both ALKBH5 and HSPB1 are known to be highly expressed in HCC and contribute to tumor progression [22–24], underscoring their significance in HCC pathogenesis. Taken together, these results suggest that combination treatment may enhance the ferroptosis in tumor tissues from a subcutaneous Hepa1-6 tumor model via modulating Alkbh5/Hspb1 axis. These findings in the current research provides a novel mechanism through which combination therapy suppressed the progression of HCC.

## Materials and methods

### Animal study

BALB/c mice (male, 20 ± 2 g, 6-week-old) were obtained from SiPeiFu (Beijing) and were fed for one week adaptively. All animals were randomly assigned for eleven groups respectively: I: control, RT (8 Gy), anti-PD-1 (Camrelizumab, a PD-1 inhibitor) and RT + anti-PD-1 groups (*n* = 6 per group); II: control, RT + anti-PD-1, si-Hspb1, and RT + anti-PD-1 + si-Hspb1 groups (*n* = 5 per group); III: control, RT + anti-PD-1 and RT + anti-PD-1 + si-Hspb1 groups (*n* = 5 per group).

Hepa1-6 cells (1 × 10^6^ cells) were injected subcutaneously into the right axilla of each mouse. Mice in the RT treatment groups were received an irradiation dose of 8 Gy for three consecutive days (Fig. S1A). Mice in the anti-PD-1 treatment groups were given tail vein injection of camrelizumab (Jiangsu Hengrui Pharmaceutical Co., LTD) once every three days (Fig. S1A). Mice in the si-Hspb1 treatment group were given tail vein injection of lentivirus-expressed si-Hspb1 (HANBIO) (Fig. S1B and S1C). Tumor volume was measured via a formula (volume = 0.5 × width × width × length). After treatment, the mice were sacrificed by cervical dislocation after isoflurane-inhalation anesthesia. Next, the tumors, serum samples, or peripheral blood samples were collected. The concentrations of alkaline phosphatase (ALP), creatinine (CREA), and blood urea nitrogen (BUN) in serum samples of mice were evaluated by an automatic biomedical analyzer (BS-240vet, Mindray). The measurements were performed by a researcher blinded to the treatment.

The animal experimental protocols were approved by the Ethics Committee of Inner Mongolia Medical University (Ref No. YKD202401160) and conducted in accordance with the National Institutes of Health Guidelines for the Care and Use of Laboratory Animals and carried out in accordance with the ARRIVE guidelines.

### H&E staining assay

Tumor tissues were fixed in 4% paraformaldehyde for 24 h and then embedded in paraffin. Next, paraffin-embedded samples were cut into 5-μm thick slices. After that, the sections were dewaxed and then stained with hematoxylin and eosin dye. A light microscope (OLYMPUS) was used to observe the pathological changes of tumor tissues.

### TUNEL staining assay

Cell apoptosis in tumor tissues was evaluated using the TUNEL cell apoptosis detection assay kit (BOSTER) according to the manufacturer’s instructions. The results were captured using a fluorescence microscope (SOPTOP OD630K, SUNNY HENPINGINSTRUMENT).

### Reverse transcription quantitative PCR (RT-qPCR)

RNA was extracted using the Redzol reagent (SBS Genetech Co., Ltd) and then reverse-transcripted into the cDNAs using the SureScript™ First-Strand cDNA Synthesis Kit (GeneCopeia). The quantitative PCR was performed using the 2×SYBR Green qPCR Master Mix (None ROX) kit (Servicebio) on a iQ5 Real-Time PCR instrument (Applied Biosystems). Relative gene expression was normalized to GAPDH or β-actin. The primers were as follows: Gpx4, 5′-GCCGTCTGAGCCGCTTACTT-3′ (forward) and 5′-TATCGGGCATGCAGATCGAC-3′ (reverse); Acsl4, 5′-CCTTCCTCTTAAGGCCGGGA-3′ (forward) and 5′-TCTTTGCCATAGCGTTTTTCTTAG-3′ (reverse); Tfr1, 5′-TGTCACTAGGCTCTCAGGGT-3′ (forward) and 5′-TGAAGACTGAAGGGAGTAGCAA-3′ (reverse); Pcbp1, 5′-AACCAGGTGGCAAGACAACA-3′ (forward) and 5′-GCAGCCGAGCCAGTAATAGT-3′ (reverse); Gapdh, 5′-AGGTCGGTGTGAACGGATTTG-3′ (forward) and 5′-GGGGTCGTTGATGGCAACA-3′ (reverse); Hspb1, 5′-ATGAGTGGTCGCAGTGGTTC-3′ (forward) and 5′-TTCGTGCTTGCCAGTGATCT-3′ (reverse); Alkbh5, 5′-GGACCACCAAGCGGAAAT-3′ (forward) and 5′-GCCCTCGCCGAAGAAGTA-3′ (reverse); Fto, 5′-GTGTCGGAACCTGTGCTTT-3′ (forward) and 5′-CTTGCGGTGGGACCTTTT-3′ (reverse); Mettl3: 5′-GACTCTGGGCACTTGGAT-3′ (forward) and 5′-ATCAGTGGGCAAGGTCAA-3′ (reverse); Mettl13: 5′-CAGAGCCCAGAAGAAACG-3′ (forward) and 5′-AGACCCACCACCAGCAAT-3′ (reverse); β-actin: 5′-GTGACGTTGACAATCCGTAAAGA-3′ (forward) and 5′-GCCGGACTCATCGTACTCC-3′ (reverse).

### Methylated RNA immunoprecipitation sequencing (MeRIP-seq)

RNA samples were isolated from tumor tissues using the Redzol reagent. After fragmentation, RNA samples were divided into two parts: IP and Input parts. The IP part of RNA samples was incubated with an anti-m6A antibody together with Dynabeads. Next, m6A antibody-combined methylated RNA was eluted from the m6A-Dynabeads for constructing MeRIP Library. The Input part of RNA samples was used for constructing regular transcriptome sequencing library. Thereafter, sequencing was performed on these two constructed libraries (m6A-seq library and RNA-sequencing library) on the Illumina NovaSeq 6000 sequencing platform. Differentially m6A peaks between two groups were screened using the MeTDiff software in R package with |log2FoldChange | ≥1 and *P* value < 0.05 as the thresholds [25]. The peaks were annotated by using the ChIPseeker software [26]. Differentially expressed genes (DEGs) between two groups were screened using DESeq2 software with |log2FoldChange | ≥1 and *P* value < 0.05 as the thresholds. Correlation analysis was then performed on the MeRIP-Seq data and transcriptome data. Next, GO and KEGG functional enrichment analyses were performed on genes that were dysregulated both at transcription and m6A methylation levels between two groups. Furthermore, MeRIP-qPCR was employed to assess m6A levels in tumor tissues, with RNA enrichment quantified by qRT-PCR.

### Cell culture and transfection

Mouse liver cancer Hepa1-6 cells were obtained from Procell Life Science & Technology Co,.Ltd (CL-0105) with STR authentication. Cells were incubated in Dulbecco’s Modified Eagle’s Medium (DMEM, Gibco) containing 10% fetal bovine serum (FBS, Gibco) at 37 °C in 5% CO_2_.

The pcDNA3.1-Hspb1 (Hspb1-OE) and pcDNA3.1-Alkbh5 (Alkbh5-OE) plasmids and siRNA (si) specifically targeting Hspb1 (si-Hspb1) were constructed by HANBIO. Additionally, lentivirus-mediated si-Hspb1 pladmids were constructed by HANBIO. Hepa1-6 cells were transfected with Hspb1-OE, si-Hspb1, Alkbh5-OE or lentivirus-expressed si-Hspb1 using the Lipo2000, respectively.

### Colony formation assay

Hepa1-6 cells were added into 12-well plates, and then cultured for 2 weeks. Next, the colonies were fixed with 4% paraformaldehyde and then stained with 0.1% crystal violet. After that, the colonies were captured using a digital camera and the number of colonies that contained ≥50 cells per colony was counted under the naked eye.

### Flow cytometry assay

The annexin V-FITC/PI apoptosis kit was used to detect the apoptosis in Hepa1-6 cells. Cells were suspended in 500 µL of binding buffer and then stained with 5 µL of Annexin V-FITC reagent and 10 µL of PI reagent in darkness at room temperature for 20 min. Next, the apoptotic cells were analyzed on a BD FACSCalibur^TM^ Flow Cytometer.

Peripheral blood samples from mice were prepared as single-cell suspensions. Single-cell suspensions were then incubated with fluorescence-conjugated antibodies targeting CD45, CD3, CD4, CD8a, CD44, CD62L, and IFN-γ at 4 °C for 1 h in the dark. The samples were then analyzed using the BD FACSCelestaTM flow cytometer (BD Biosciences, CA).

### Wound healing assay

Hepa1-6 cells were seeded into 6-well plates. When cells reached 90-95% confluence, the cell monolayer was scratched with a with a 200 µl sterile pipette tip. After washing three times with PBS, the detached cells were removed. Next, cells were cultured for 0, 24 and 48 h at 37 °C in 5% CO_2_. The results were then observed using a microscope (OLYMPUS).

### Transmission electron microscopy (TEM)

The samples were fixed in 2.5% glutaraldehyde (pH 7.4) for 2 h and then fixed in 1% osmic acid at 4 °C for 2 h. After gradient dehydration, the samples were embedded in Epon-Araldite resin. and then the ultrathin sections were made. The ultrathin sections were counterstained with 3% uranyl acetate and 2.7% lead citrate. Finally, a HT7800 transmission electron microscope was utilized to observe the ultrastructure of the samples.

### Detection of reactive oxygen species (ROS)

A ROS detection kit (E004-1-1, Nanjing Jiancheng) was used to evaluate intracellular ROS level. Cells were probed with 2,7-Dichlorofluorescin diacetate (DCFH-DA) for 30 min at 37 °C. A fluorescence microscope was then used to observe the results.

### Measurement of iron content

The intracellular Fe2+ concentration in cells and tumor tissues were detected using the Ferrous Iron Colorimetric Assay Kit (E-BC-K773-M, Elabscience) according to the manufacturer’s instructions. Next, the results were measured by a microplate reader (Bio-tek).

### Western blot assay

Protein concentration was measured using the Bradford protein concentration detecting kit (BOSTER Biological Technology co. Ltd.). Next, protein was separated by polyacrylamide gels (10%), transferred onto polyvinylidene difluoride membranes. Membranes were then immunoprobed overnight at 4 °C with anti-Alkbh5 (Monoclonal antibody, 67811-1-Ig, Proteintech), anti-Hspb1 (Polyclonal antibody, 18284-1-AP, Proteintech), anti-Tfr1 (Polyclonal antibody, 10084-2-AP, Proteintech), anti-Pcbp1 (Monoclonal antibody, ab168378, Abcam), anti-Gpx4 (Polyclonal antibody, 30388-1-AP, Proteintech), anti-Slc7a11 (Polyclonal antibody, 26864-1-AP, Proteintech), anti-Acsl4 (Polyclonal antibody, 22401-1-AP, Proteintech), anti-Gapdh (Polyclonal antibody, 10494-1-AP, Proteintech) and anti-β-actin (Polyclonal antibody, ab8227, Abcam) antibodies, followed by incubation with a secondary antibody. The blots were developed by the chemiluminescence.

### Metabolomics analysis

For sample pretreatment, methanol-acetonitrile (v:v = 2:1) solution were added into the serum samples collected from mice. Samples in the 1.5 mL eppendorf tube were then extracted in an ice-water bath in an ultrasonic instrument for 10 min, and maintained at –40 °C overnight. Next, samples were centrifuged at 12,000 rpm for 10 min at 4 °C and the supernatant was put into the LC-MS autosampler vial. After volatilization of liquid, samples were re-dissolved in methanol-water (v:v = 1:4), and then extracted in an ice-water bath in an ultrasonic instrument for 3 min. After standing at -40 °C for 2 h, samples were centrifuged at 12000 rpm at 4 °C. After that, the supernatant was filtered through a 0.22 µm filter and then loaded into the LC-MS autosampler vial. Samples were stored at –80 °C. The samples were subjected to liquid chromatography-mass spectrometry (LC-MS) analysis on a Waters ACQUITY UPLC I-Class plus/Thermo QE plus instrument.

Principal component analysis (PCA), partial least squares discrimination analysis (PLS-DA) and orthogonal partial least square discriminant analysis (OPLS-DA) were conducted to determine the differences in metabolic profiles among three groups. Differential metabolites between two groups were screened by using OECloud tools (https://cloud.oebiotech.com) with variable importance in the projection (VIP) > 1 and *P*value < 0.05 as the thresholds. Next, KEGG was performed to analyze the potential pathways involved in these differential metabolites.

### Statistical analysis

Data were expressed as means ± standard deviation. Differences among multiple groups were analyzed using the one-way ANOVA followed by Tukey’s test. All experiments were performed at least in triplicates. *P* < 0.05 was regarded as statistically significant.

## Results

### Combination of RT and IO inhibited the progression of murine HCC in vivo

**Fig. 1:**
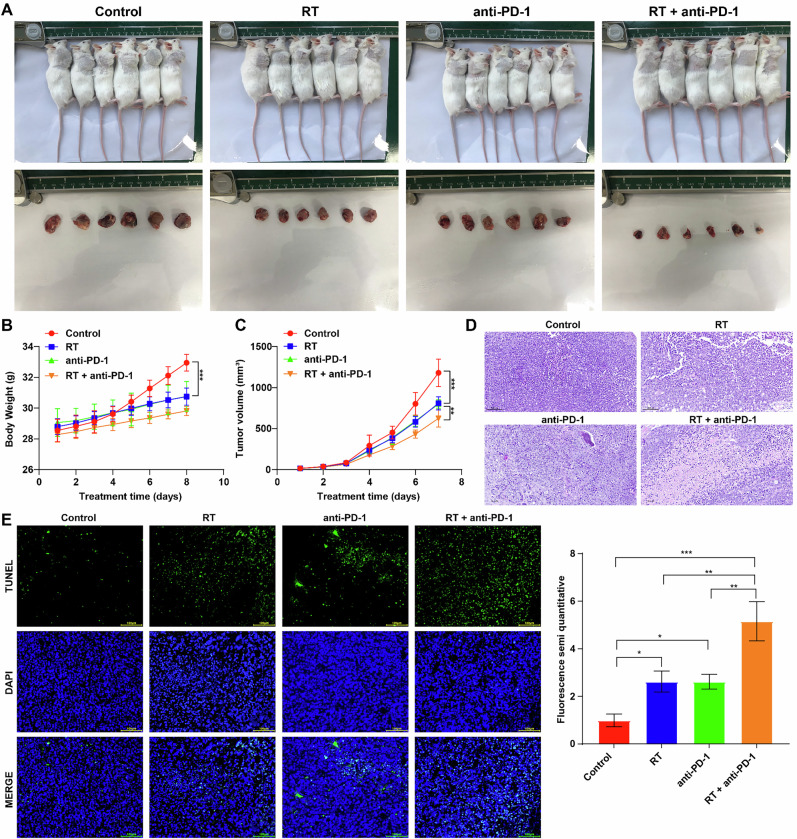
Combination of radiotherapy and immunotherapy inhibits the progression of murine HCC in vivo. **A** Photograph of the Hepa1-6 tumor-bearing mice (upper panel) and corresponding tumor tissues collected from mice (lower panel). **B** The body weight of mice in different groups. **C** Tumor volume of mice in different groups. **D** H&E staining assay was performed to observe pathological changes of tumor tissues. **E** The cell apoptosis in tumor tissues was determined using TUNEL staining assay. **P < 0.01, ***P < 0.001.

A Hepa1-6 cell mouse model was established to explore the anti-tumor effects of RT and anti-PD-1 in vivo. As shown in Fig. 1A–C, compared to control group, RT, anti-PD-1 or combination therapy significantly reduced body weights and tumor volume of mice. Additionally, compared to the RT or anti-PD-1 single treatment group, combination therapy further lowered tumor volume of mice (Fig. 1A, C). Moreover, H&E staining results showed that compared to the single treatment group, a large area of tumor necrosis were observed in the RT + anti-PD-1 treatment group (Fig. 1D). Meanwhile, the results of TUNEL staining assay indicated that both RT and anti-PD-1 single treatment notably induced the apoptosis in tumor tissues (Fig. 1E). As expected, combination therapy further triggered cell apoptosis in tumor tissues compared to the single treatment group (Fig. 1E). To sum up, combination therapy could inhibit murine HCC progression in vivo. Furthermore, the serum levels of ALP, CREA and BUN in mice from the RT + anti-PD-1 treatment group were increased compared to the control group but remained within normal physiological ranges [27, 28] (Fig. S2A–C).

### Combination of RT and IO increased m6A modification of Hspb1 mRNA and reduced Hspb1 mRNA level in murine HCC model

**Fig. 2:**
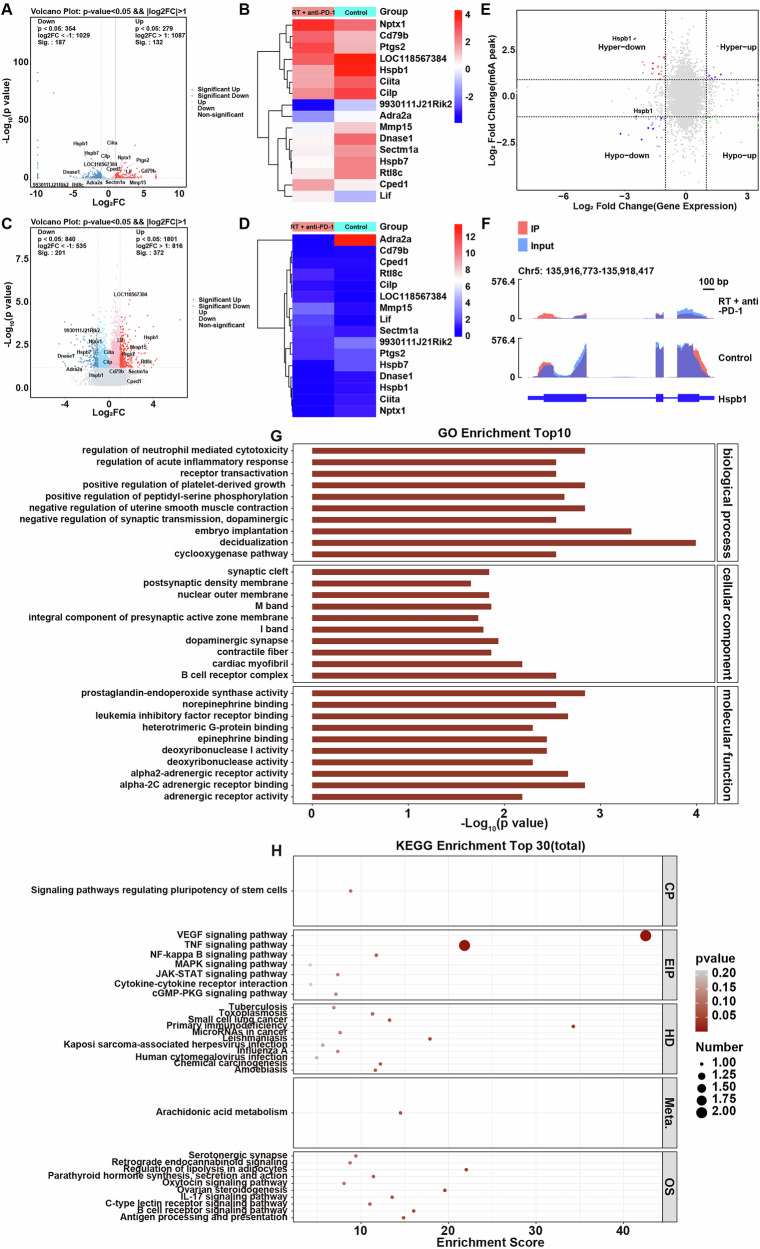
Combination of radiotherapy and immunotherapy increased m6A modification of Hspb1 mRNA and reduced Hspb1 mRNA level in murine HCC model. **A** Volcano map showed differentially expressed genes (DEGs) between control and RT + anti-PD-1 groups. **B** Heatmaps of several major DEGs between control and RT + anti-PD-1 groups. **C** Volcano map showed differentially methylated genes (DMGs) between control and RT + anti-PD-1 groups. **D** Heatmaps of several major DMGs between control and RT + anti-PD-1 groups. **E** Four-quadrant scatter plot of transcriptome and methylation levels using MeRIP-seq data. **F** IGV plot showing m6A methylation profiles for Hspb1 mRNA. **G**, **H** GO and KEGG analyses were performed on the common genes between DEGs and DMGs.

To explore the anti-tumor mechanism of combination of RT and IO in HCC, MeRIP-seq was performed to screen genes modified by m6A methylation in HCC in vivo between control and RT + anti-PD-1 groups. A total of 319 DEGs, including Nptx1, Cd79b, Ptgs2, LOC118567384, Hspb1, Cilta, Cilp, 9930111J21Rik2, Adra2a, Mmp15, Dnase1, Sectm1a, Hspb7, Rtl8c, Cped1 and Lif, were screened in tumor tissues between two groups (Fig. 2A, B). Additionally, compared to the control group, a total of 573 differentially methylated genes (DMGs) exhibited lower or higher levels of m6A modification in tumor tissues in the RT + anti-PD-1 group (Fig. 2C, D). According to the results of correlation analysis between transcriptome and methylation level, the m6A abundance in Hspb1 mRNA was notably increased, whereas Hspb1 mRNA level was markedly reduced in the RT + anti-PD-1 group, compared to the control group (Fig. 2E). Meanwhile, IGV visualization results showed that the m6A levels on Hspb1 gene in tumor tissues were significantly increased in tumor tissues from tumor-bearing mice after combination therapy, compared to the control group (Fig. 2F). Furthermore, meRIP-qPCR analysis revealed that the m6A modification level of Hspb1 in tumor tissues was significantly higher in both the RT and anti-PD-1 groups compared to the control group (Fig. S3). Notably, the combination treatment group exhibited a further increase in Hspb1 m6A modification compared to either single treatment alone (Fig. S3). Next, GO and KEGG analyses were performed on genes that were dysregulated both at transcription and m6A methylation levels between two groups. GO analysis showed that these overlapping genes were enriched in 282 GO terms such as “lipid metabolic process”, “negative regulation of lipid catabolic process” (Fig. 2G and Table S1). KEGG analysis showed that these genes were related to 35 KEGG pathways including “Arachidonic acid metabolism”, “Regulation of lipolysis in adipocytes”, “TNF signaling pathway” and “NF-kappa B signaling pathway” (Fig. 2H and Table S2).

### Modulation of Hspb1 expression level impacted Hepa1-6 cell proliferation, migration, and apoptosis

**Fig. 3:**
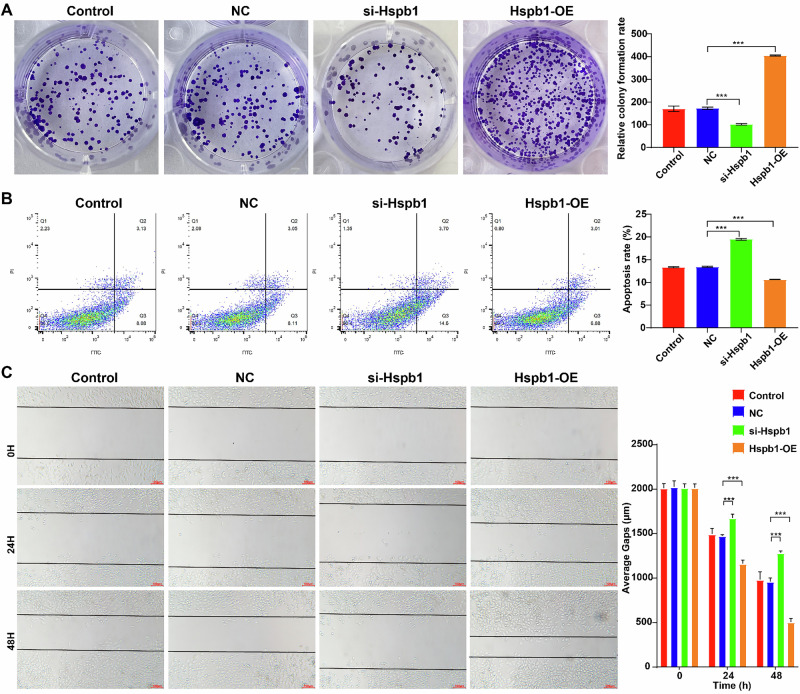
Modulation of Hspb1 expression level impacted Hepa1-6 cell proliferation, migration, and apoptosis. Hepa1-6 cells were transfected with si-Hspb1 or Hspb1-OE. **A** Colony formation assay was conducted to assess cell proliferation. **B** Flow cytometry was applied to evaluate cell apoptosis. **C** Wound healing assay was performed to assess cell migratory ability. ***P < 0.001.

Next, we sought to explore the role of Hspb1 on Hepa1-6 cells. The results showed that downregulation of Hspb1 remarkably suppressed Hepa1-6 cell proliferation and migration and triggered cell apoptosis (Fig. 3A–C). Conversely, overexpression of Hspb1 obviously enhanced Hepa1-6 cell proliferation and migration and reduced Hepa1-6 cell apoptosis (Fig. 3A–C). Collectively, Hspb1 could affect Hepa1-6 cell growth.

### Modulation of Hspb1 expression level impacted the ferroptosis of Hepa1-6 cells

**Fig. 4:**
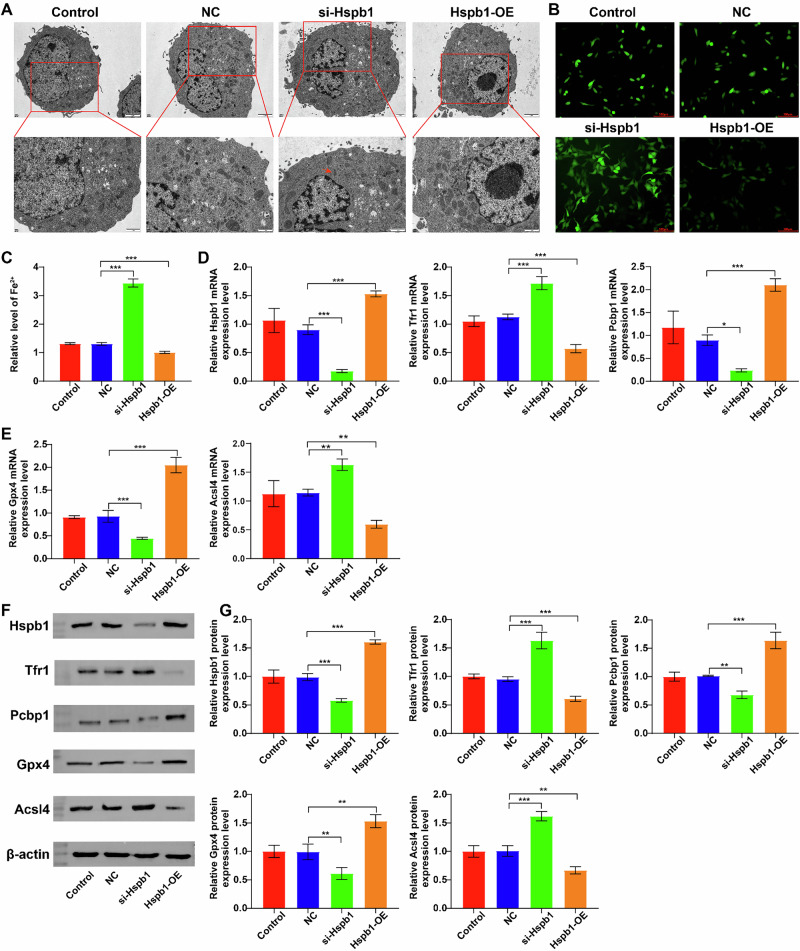
Modulation of Hspb1 expression level impacted the ferroptosis of Hepa1-6 cells. Hepa1-6 cells were transfected with si-Hspb1 or Hspb1-OE. **A** The morphological changes of cells were examined using TEM (red arrow, the disappearance of mitochondrial cristae). **B** Intracellular ROS level was evaluated by the DCFH-DA staining assay. **C** The content of Fe^2+^ in cells were detected by an ELISA kit. **D–G** RT-qPCR and western blot assays were performed to measure Hspb1, Tfr1, Pcbp1, Gpx4 and Acsl4 levels in Hepa1-6 cells. *P < 0.05, **P < 0.01, ***P < 0.001.

We next employed TEM to evaluate the effect of Hspb1 on the ultrastructure of Hepa1-6 cells. As shown in Fig. 4A, Hspb1 downregulation obviously caused the disappearance of mitochondrial cristae, a morphological hallmark consistent with ferroptosis [29, 30]. Additionally, Hspb1 downregulation notably promoted ROS production and increased Fe^2+^ level in Hepa1-6 cells; however, Hspb1 overexpression displayed the opposite effects (Fig. 4B, C). Moreover, Hspb1 downregulation remarkably reduced Hspb1 level and decreased Pcbp1 and Gpx4 levels and increased Tfr1 and Acsl4 levels in Hepa1-6 cells; however, Hspb1 overexpression displayed the opposite effects (Fig. 4D–G). Collectively, Hspb1 could affect the ferroptosis of Hepa1-6 cells.

### Combination of RT and IO promoted the ferroptosis of Hepa1-6 cells in vitro through inhibiting Alkbh5/Hspb1 axis

**Fig. 5:**
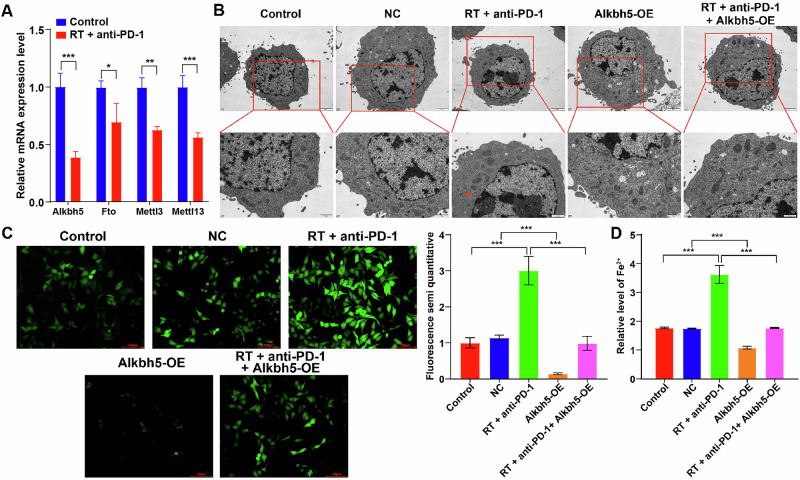
Combination of radiotherapy and immunotherapy promoted the ferroptosis of Hepa1-6 cells in vitro through inhibiting Alkbh5/Hspb1 axis. **A** RT-qPCR was performed to determine Alkbh5, Fto, Mettl3 and Mettl13 levels between control and RT + anti-PD-1 groups in murine HCC model. **B** Hepa1-6 cells were treated with RT+anti-PD-1 or/and Alkbh5-OE. The morphological changes of cells were examined using TEM (red arrow, the disappearance of mitochondrial cristae). **C** Intracellular ROS level was evaluated by the DCFH-DA staining assay. **D** The content of Fe^2+^ in cells were detected by an ELISA kit. *P < 0.05, **P < 0.01, ***P < 0.001.

**Fig. 6:**
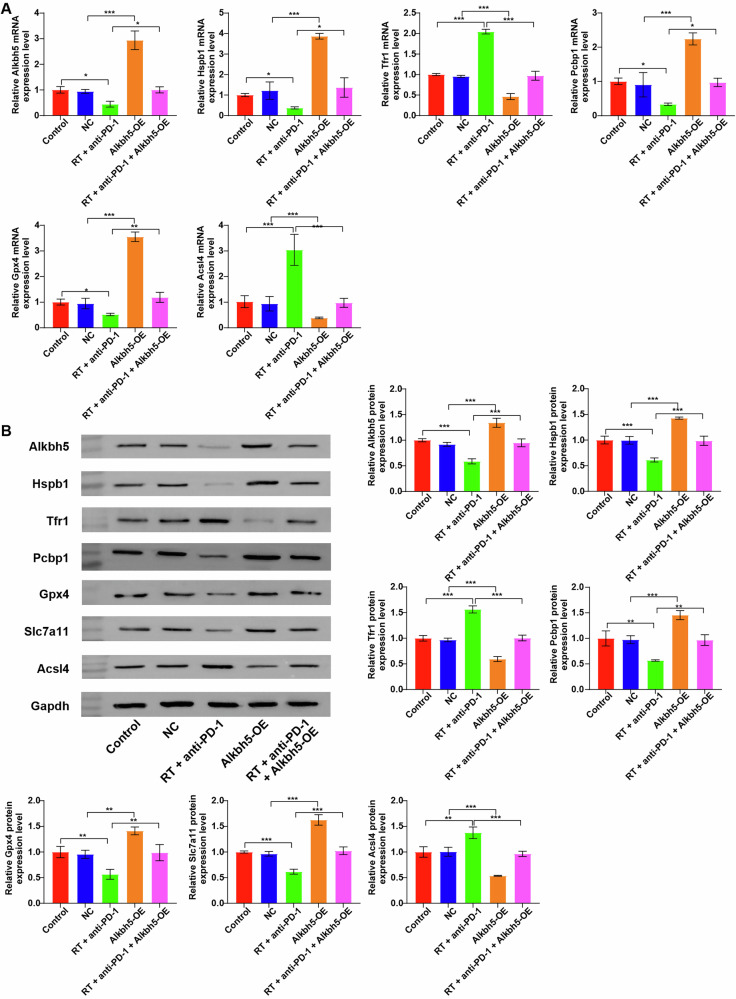
Combination of radiotherapy and immunotherapy impacted the expression levels ferroptosis-related proteins in Hepa1-6 cells through targeting Alkbh5/Hspb1 axis. Hepa1-6 cells were treated with RT+anti-PD-1 or/and Alkbh5-OE. **A** RT-qPCR assay was performed to measure Alkbh5, Hspb1, Tfr1, Pcbp1, Gpx4, and Acsl4 levels in Hepa1-6 cells. **B** Western blot assay was performed to measure Alkbh5, Hspb1, Tfr1, Pcbp1, Gpx4, Slc7a11 and Acsl4 levels in Hepa1-6 cells. *P < 0.05, **P < 0.01, ***P < 0.001.

We have known that the m6A level of Hspb1 mRNA was dysregulated between control and RT + anti-PD-1 group (Fig. 2). Then, we performed RT-qPCR to assess the expression of m6A-related enzymes in the control and RT + anti-PD-1 groups to investigate whether the dysregulation of Hspb1 is associated with changes in these enzymes. As shown in Fig. 5A, treatment with RT + anti-PD-1 markedly suppressed the expression of the methyltransferases Mettl3 and Mettl13 as well as the demethylases Alkbh5 and Fto, with the most notable downregulation observed in Alkbh5 expression. Alkbh5, a key RNA demethylase, has been found to exert important roles in cancer development [31, 32]. Thus, we next to explore whether combination treatment could induce the ferroptosis of Hepa1-6 cells, and whether Alkbh5 participated in the pro-ferroptotic effect of combination treatment in Hepa1-6 cells through targeting Hspb1. As shown in Fig. 5B, combination treatment obviously resulted in mitochondria damage in Hepa1-6 cells, characterized by the disappearance of mitochondrial cristae. Additionally, combination treatment significantly elevated ROS production and Fe^2+^ level in Hepa1-6 cells, whereas these changes were abolished by Alkbh5 overexpression (Fig. 5C, D). Moreover, compared to control group, combination treatment markedly decreased Alkbh5, Hspb1, Pcbp1, Gpx4, Slc7a11 levels and increased Tfr1 and Acsl4 levels in Hepa1-6 cells, whereas these phenomena were reversed by Alkbh5 overexpression (Fig. 6A, B). To sum up, combination therapy could promote the ferroptosis of Hepa1-6 cells in vitro through inhibiting Alkbh5/Hspb1 axis.

### Combination of RT and IO promoted the ferroptosis in tumor tissues in vivo through targeting Alkbh5/Hspb1 axis

**Fig. 7:**
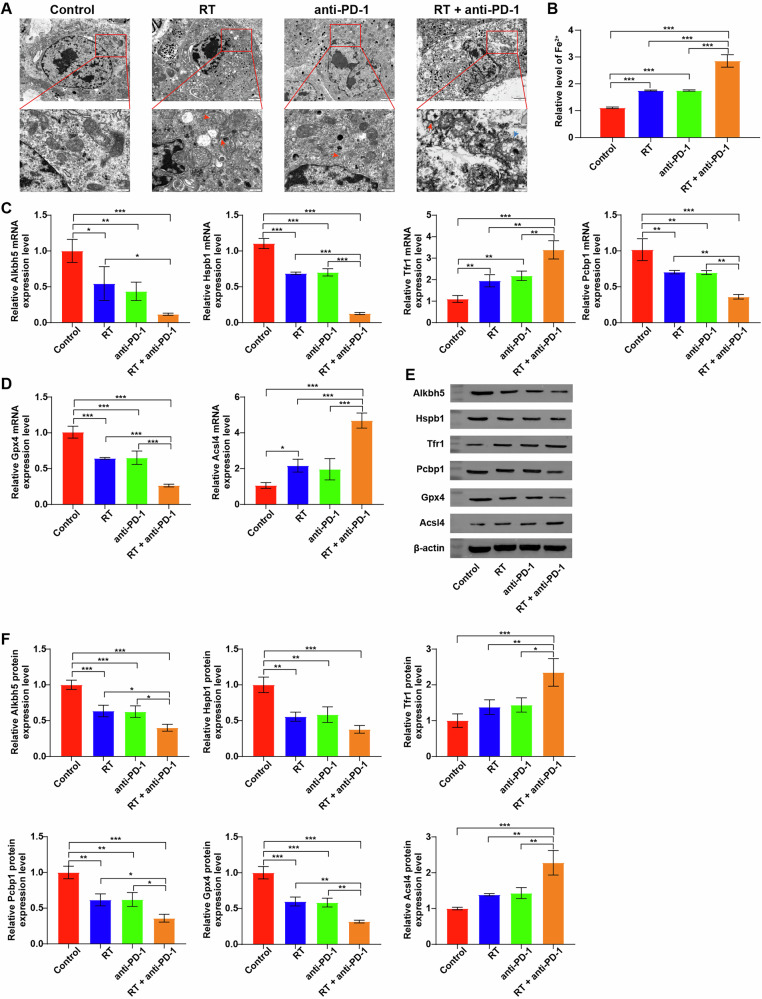
Combination of radiotherapy and immunotherapy promoted the ferroptosis of Hepa1-6 cells in vivo through targeting Alkbh5/Hspb1 axis. **A** The morphological changes of tumor tissues from Hepa1-6 tumor-bearing mice were examined using TEM (red arrow, the disappearance of mitochondrial cristae; blue arrow, mitochondrial outer membrane rupture). **B** The content of Fe^2+^ were detected by an ELISA kit. **C**–**F** RT-qPCR and western blot assays were performed to measure Alkbh5, Hspb1, Tfr1, Pcbp1, Gpx4 and Acsl4 levels in tumor tissues. *P < 0.05, **P < 0.01, ***P < 0.001.

Next, we examined the effect of RT + anti-PD-1 on the ferroptosis of tumor tissues in a Hepa1-6 cell mouse model in vivo. As shown in Fig. 7A, combination therapy greatly lead to abnormal mitochondrial changes, marked by cristae disappearance and outer membrane rupture. Moreover, compared to the single treatment group, combination therapy notably elevated Fe2+ level in serum samples of tumor-bearing mice (Fig. 7B). Meanwhile, compared to the single treatment group, RT + anti-PD-1 treatment further decreased Alkbh5, Hspb1, Pcbp1 and Gpx4 levels and increased Tfr1 and Acsl4 levels in tumor tissues (Fig. 7C–F). To sum up, combination treatment could promote the ferroptosis in tumor tissues in vivo through targeting Alkbh5/Hspb1 axis.

### Si-Hspb1 in combination with RT and IO enhanced T-cell-mediated anti-tumor immunity in vivo

**Fig. 8:**
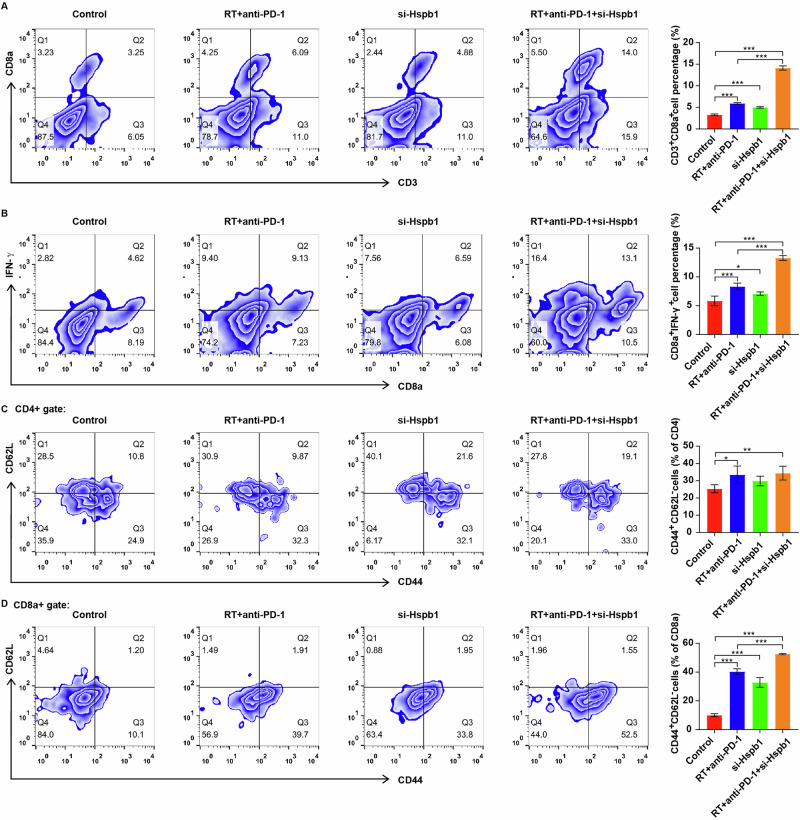
Si-Hspb1 in combination with RT and IO enhanced T-cell-mediated anti-tumor immunity in vivo. Flow cytometry analyses of the populations of (**A**) CD3 + CD8a + , **B** CD8a+ IFN-γ + , **C** CD4 + CD44 + CD62L- and **D** CD8a + CD44 + CD62L- T cells in the peripheral blood samples from Hepa1-6 tumor-bearing mice. *P < 0.05, **P < 0.01, ***P < 0.001.

Next, we explored the percentage of cytotoxic T cells (CD3 + CD8a + ), IFN-γ-expressing cytotoxic T cells (CD8a+IFN-γ + ), and CD4+ and CD8+ effector memory T cells (CD4 + CD44 + CD62L-; CD8a + CD44 + CD62L-) in the peripheral blood samples from tumor-bearing mice. As shown in Fig. 8A–D, RT + anti-PD-1 treatment significantly elevated the percentage of these T cell subpopulations in the peripheral blood samples of tumor-bearing mice. Meanwhile, downregulation of Hspb1 further increased the percentage of CD3 + CD8a + , CD8a+ IFN-γ+ and CD8a + CD44 + CD62L- T cells in the peripheral blood samples in vivo compared to the RT + anti-PD-1 group, but not CD4 + CD44 + CD62L- T cells (Fig. 8A–D). Collectively, these results suggested that si-Hspb1 in combination with RT and IO was able to enhance T cell activation and strengthen anti-tumor immunity in vivo.

### Screening differentially metabolites among the three groups

**Fig. 9:**
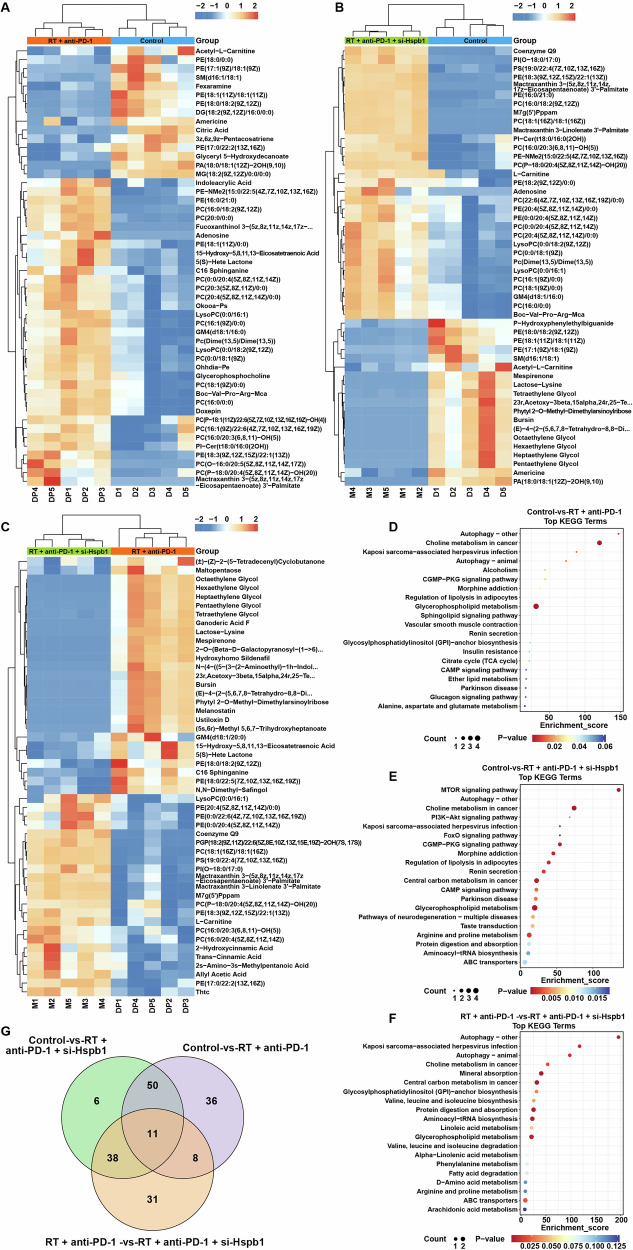
Screening differentially metabolites among the three groups. Heatmap showed top 50 differentially metabolites between (**A**) control and RT + anti-PD-1 groups, **B** control and RT + anti-PD-1 + si-Hspb1 groups, **C** between RT + anti-PD-1 and RT + anti-PD-1 + si-Hspb1 groups. KEGG analysis on differentially metabolites between (**D**) control and RT + anti-PD-1 groups, **E** control and RT + anti-PD-1 + si-Hspb1 groups, **F** between RT + anti-PD-1 and RT + anti-PD-1 + si-Hspb1 groups. **G** Venn diagram showing 11 common differential metabolites among three groups.

To further verify the pro-ferroptosis role of combination therapy in HCC, we explored whether lipid metabolism abnormalities were appeared across control, RT + anti-PD-1, and RT + anti-PD-1 + si-Hspb1 groups using the untargeted metabolomic profiling. The results of PCA, PLS-DA, and OPLS-DA showed obvious differences in the distributions of the samples from control, RT + anti-PD-1, or RT + anti-PD-1 + si-Hspb1 groups, suggesting a significant difference of metabolites among three groups (Fig. S4A–C). A total of 105, 105, 88 significantly differentially metabolites were screened between control and RT + anti-PD-1 groups, between control and RT + anti-PD-1 + si-Hspb1 groups, and between RT + anti-PD-1 and RT + anti-PD-1 + si-Hspb1 groups, respectively (Fig. 9A–C, Fig. S5–S8 and Table S3). The results of KEGG enrichment pathway analysis showed that some of these metabolites were found to participate in glycerophospholipid metabolism pathway, a pathway belongs to lipid metabolism (Fig. 9D–F, Tables S4, S5, S6).

A Venn diagram was performed to screen common differentially metabolites among all groups. The results showed that there were 11 differentially metabolites among control, RT + anti-PD-1 or RT + anti-PD-1 + si-Hspb1 groups (Fig. 9G and Table S7). Among these 11 metabolites, three glycerophospholipids metabolites including PE(18:1(11Z)/0:0), PE(18:3(9Z,12Z,15Z)/22:1(13Z)) and PI(O-18:0/17:0) were increased in RT + anti-PD-1 and RT + anti-PD-1 + si-Hspb1 groups compared to the control group, respectively (Table 1). Meanwhile, compared to RT + anti-PD-1 group, these three glycerophospholipids metabolites were further increased in RT + anti-PD-1 + si-Hspb1 group (Table 1).

**Table 1:** List of 11 common differential metabolites identified in three groups.

No.	Metabolites	m/z	Retention time (min)	Ion mode	RT + PD-1 vs. control	RT + anti-PD-1 + si-Hspb1 vs. control	RT + anti-PD-1 + si-Hspb1 vs. RT + PD-1	Metabolic pathway	
								Class	Sub Class
1	LysoPC(0:0/16:1)	494.3240	10.14	pos	↑	↑	↑	Unclassified	Unclassified
2	3z,6z,9z-Pentacosatriene	369.3512	10.53	pos	↓	↓	↓	Unsaturated hydrocarbons	Olefins
3	**PE(18:1(11Z)/0:0)**	480.3085	10.85	pos	**↑**	**↑**	**↑**	**Glycerophospholipids**	**Glycerophosphoethanolamines**
4	PE(17:0/22:2(13Z,16Z))	830.5925	10.58	neg	↓	↑	↑	Unclassified	Unclassified
5	PE(18:0/18:2(9Z,12Z))	744.5533	12.56	pos	↓	↓	↓	Glycerophospholipids	Glycerophosphoethanolamines
6	PC(16:0/20:3(6,8,11)-OH(5))	782.5670	12.56	pos	↑	↑	↑	Unclassified	Unclassified
7	PC(P-18:0/20:4(5Z,8Z,11Z,14Z)-OH(20))	832.5820	13.12	pos	↑	↑	↑	Unclassified	Unclassified
8	**PE(18:3(9Z,12Z,15Z)/22:1(13Z))**	818.5647	13.10	pos	**↑**	**↑**	**↑**	**Glycerophospholipids**	**Glycerophosphoethanolamines**
9	Mactraxanthin 3-(5z,8z,11z,14z,17z-Eicosapentaenoate) 3’-Palmitate	1197.8469	12.28	pos	↑	↑	↑	Prenol lipids	Tetraterpenoids
10	**PI(O-18:0/17:0)**	821.5866	13.29	pos	**↑**	**↑**	**↑**	**Glycerophospholipids**	**Glycerophosphoinositols**
11	Mactraxanthin 3-Linolenate 3’-Palmitate	1173.8466	13.35	pos	↑	↑	↑	Prenol lipids	Tetraterpenoids

↑, shows upregulated metabolites compared with control or RT + anti-PD-1 group.

↓, shows dwonregulated metabolites compared with control or RT + anti-PD-1 group.

## Discussion

A combination of RT and IO is now becoming a prospective method for the treatment of multiple cancers [33]. Mechanically, RT could enhance systemic response to IO through promoting antigen presentation, recruiting tumor specific T cells into tumor tissues, and elevating PD-L1 level in tumor tissues [34, 35]. For instance, Philippou et al. found that RT could alter the tumor immune microenvironment in a murine prostate cancer model through elevating the proportion of dendritic cells and CD4 + T cells and upregulation of PD-1/PD-L1 axis [36]. Furthermore, Du et al. have demonstrated the critical role of CD8+ cytotoxic T cells in mediating the synergistic antitumor effects of RT combined with anti-PD-L1 therapy in HCC [37]. In the current research, we for the first time found that combination of RT and anti-PD-1 treatment could exert anti-tumor effects in HCC through enhancing the ferroptosis. This finding provides new insights into the molecular mechanisms underlying RT-IO combination therapy, revealing an additional tumor cell-intrinsic death pathway that complements the known immune-mediated effects.

Evidence have shown that ferroptosis is an iron-dependent form of cell death that is triggered by lipid peroxidation [38], contributes to the anti-tumor role of RT and IO in human cancers [39, 40]. Ye et al. indicated that RT was able to induce cancer cell ferroptosis, with ferroptosis inducers (e.g., imidazole ketone erastin) enhancing RT efficacy [41]. Furthermore, Wang et al. reported an anti-tumor mechanism during cancer IO, the results showed that CD8 + T cell-derived IFN-γ could reduce SLC3A2 and SLC7A11 (two ferroptosis inhibitors) levels, thereby leading to lipid peroxidation and ferroptosis in tumor cells [42]. Moreover, enhancing ferroptosis also can boost antitumor immunity through impairing Treg cell homeostasis [43]. These results showed a close relationship between ferroptosis and antitumor immunity. Notably, it’s worth noting that IO treatment could sensitize tumors to RT through enhancing tumor cell ferroptosis [39]. In out study, we found that compared to RT or anti-PD-1 single treatment, combination therapy notably inhibited murine HCC progression in vivo. Moreover, combination treatment obviously elevated intracellular Fe2+ level, as well as increased Acsl4 level and reduced Gpx4 level in tumor tissues from tumor-bearing mice with HCC, compared to the single treatment group. These results showed that combination therapy could inhibit HCC progression via inducing ferroptosis.

Evidence has shown that aberrant m6A modification is closely related to the tumorigenesis and progression of cancers [44]. Meanwhile, aberrant regulation of m6A modification plays crucial roles in RT and IO [44, 45]. For example, downregulation of m6A methyltransferase METTL3 could enhance radiosensitivity or potentiate anti-tumor immunity in cancer cells [46, 47]. In the current research, we found that Hspb1, a negative regulator of ferroptosis, was dysregulated in tumor tissues between control and RT + anti-PD-1 groups. Based on the MeRIP-seq data, we found that m6A modification status of Hspb1 mRNA was increased in RT + anti-PD-1 group, compared to the control group. However, RT-qPCR results showed that compared to the control group, the mRNA level of Hspb1 was notably reduced in tumor tissues of tumor-bearing mice in the RT + anti-PD-1 group. These results showed that m6A modification may affect the mRNA level of Hspb1 in HCC. Thus, to find an upstream regulator of Hspb1 mRNA, we performed RT-qPCR to assess the expression levels of certain methyltransferases and demethylases between control and RT + anti-PD-1 groups. The results showed that the mRNA level of demethylase Alkbh5 was strongly reduced in the RT + anti-PD-1 group compared to the control group. Moreover, RT-qPCR results revealed that overexpression of Alkbh5 could notably increase Hspb1 level in murine HCC cells, suggesting that Hspb1 was a downstream target of Alkbh5. ALKBH5, as an m6A eraser, is responsible for removing m6A from target mRNA [48]. Conversely, low ALKBH5 level is related to increased m6A mRNA levels [49]. Liu et al. found that ALKBH5 could increase m6A demethylation of GLUT4 mRNA, and then elevated the mRNA level of GLUT4 in breast cancer cells [50]. Consistently, we suspected that Alkbh5 could increase Hspb1 level in murine HCC cells by mediating Hspb1 m6A demethylation. Meanwhile, RT + anti-PD-1 treatment may reduce Hspb1 level in murine HCC cells through decreasing Alkbh5-mediated m6A demethylation of Hspb1 mRNA.

HSPB1 has been recognized as a novel negative regulator of ferroptosis [51]. Liang et al. found that HSPB1 could enhance doxorubicin resistance via inhibiting ferroptosis in breast cancer [52]. In this study, we found that downregulation of Hspb1 notably elevated Fe2+ and ROS levels, and reduced Gpx4 level and increased Acsl4 level in murine HCC cells, suggesting that Hspb1 deficiency could induce ferroptosis in HCC. Moreover, overexpression of Alkbh5 obviously reversed combination treatment-induced downregulation of Hspb1 and upregualtion of Fe2+ and ROS levels in murine HCC cells. Collectively, combination treatment could induce ferroptosis in HCC by downregulating Hspb1 level via inhibiting Alkbh5-mediated m6A demethylation of Hspb1 mRNA.

To address whether lipid metabolism dysregulation links the combined therapy to ferroptosis in HCC, we performed untargeted metabolomics. Strikingly, lipid metabolites, particularly glycerophospholipids like glycerophosphoethanolamine (GPE), accounted for the majority of the differential metabolites in serum from combined therapy-treated mice (Table S3). GPE is a metabolite of phosphatidylethanolamine (PE), a major membrane phospholipid [53, 54]. Crucially, PE containing polyunsaturated fatty acids (PE-PUFAs) serves as the primary target for lipid peroxidation [55]. ACSL4 catalyzes the incorporation of PUFAs into PE to form PE-PUFAs, while iron activates lipoxygenases to peroxidize these PE-PUFAs [55, 56]. The resulting lipid peroxidation products accumulate, causing membrane damage and ultimately ferroptosis [57, 58]. Our results showed that compared to the control group, the levels of 2 GPE metabolites [PE(18:1(11Z)/0:0) and PE(18:3(9Z,12Z,15Z)/22:1(13Z))], were obviously upregulated in the serum samples of mice in the RT + anti-PD-1 group (Table 1). Notably, Hspb1 knockdown further elevated these GPE metabolites, mechanistically linking Hspb1 inhibition to amplified ferroptosis through glycerophospholipid metabolism. Collectively, these results demonstrate that the combined therapy-induced lipid metabolic shifts (particularly PE-PUFA accumulation) may directly drive ferroptotic execution, and that Hspb1 serves as a critical modulator of this process, potentiating RT/IO-induced tumor cell death.

T cells play a crucial role in mediating anti-tumor immunity, possessing immune memory function and a specific killing effect [59]. Among T cell subpopulations, CD8 + T cells are essential anti-tumor effector cells that can differentiate into cytotoxic and effector CD8 + T cells to exert their anti-tumor roles [60, 61]. Upon activation, CD8+ cells can secrete large amounts of IFN-γ to effectively eliminate cancer cells [62]. However, the interaction between PD-L1 and PD-1 could impair T cell function through inhibiting T cell cytotoxic production and T cell proliferation [63]. In this research, combination of RT and anti-PD-1 treatment could obviously increase the percentage of CD8a+ cytotoxic T cells, IFN-γ-expressing CD8a+ cytotoxic T cells, and CD4+ and CD8+ effector memory T cells, suggesting that combination treatment could potentiate the T cell (CD4+ and CD8 + T cell)-mediated anti-tumor immune. Interestingly, downregulation of Hspb1 only further increased the percentage of CD8a+ cytotoxic T cells, IFN-γ-expressing CD8a+ cytotoxic T cells, and CD8+ effector memory T cells in the peripheral blood samples of tumor-bearing mice, but not CD4+ effector memory T cells. Consequently, we conclude that the anti-tumor efficacy of si-Hspb1 was primarily dependent on CD8 + T cell-mediated immunity in tumor-bearing mice in vivo. Meanwhile, promotion of ferroptosis may further enhance RT and IO-induced anti-tumor immunity in HCC.

This study presents a limitation. Our results demonstrated modest elevations of ALP, CREA and BUN were observed in the combination therapy group compared to the control group. Although all values remained within normal physiological limits [27, 28], these findings may suggest potential systematic toxicity in clinical translation. Therefore, to address potential challenges in clinical translation, we propose several strategies for future studies aimed at minimizing potential side effects: optimizing treatment protocols to mitigate acute metabolic burden, utilizing adjunctive hepatorenal protectants during combination therapy, and implementing real-time monitoring of weight dynamics and metabolic biomarkers to facilitate adaptive treatment adjustments.

### Conclusion

In the current research, we found that RT combined with anti-PD-1 therapy could inhibit tumor growth and induce ferroptosis in murine HCC in vivo. Moreover, deficiency of Hspb1 also induced ferroptosis in murine HCC cells in vitro. Mechanistically, RT combined with anti-PD-1 could induce cell ferroptosis in HCC by downregulating Hspb1 level via reducing Alkbh5-mediated m6A demethylation of Hspb1 mRNA. Importantly, promotion of ferroptosis by Hspb1 deficiency may further enhance RT and IO-induced anti-tumor immunity in HCC. Collectively, we proposed that targeting Alkbh5/Hspb1/ferroptosis axis may enhance anti-tumor effects in combining RT with IO, highlighting a potential therapeutic approach for HCC.

## Supplementary information

supplementary figures

Table S1

Table S2

Table S3

Table S4

Table S5

Table S6

Table S7

## Data Availability

The data supporting the conclusion of this study are included in the manuscript and supplementary materials. Meanwhile, the raw MeRIP-seq data for this study has been uploaded to the OMIX database, under the accession number OMIX012286.
