# Supplementary material for: Radiotherapy combined with anti-PD-1 immunotherapy promotes ferroptosis-driven control of hepatocellular carcinoma
Source: Genes Immun. 2025 Dec 19;27(1):91–105. doi: 10.1038/s41435-025-00370-2 (PMC12923356; doi:10.1038/s41435-025-00370-2)
Supplement: Supplementary file 1 — supplementary figures [file 41435_2025_370_MOESM1_ESM.pdf]

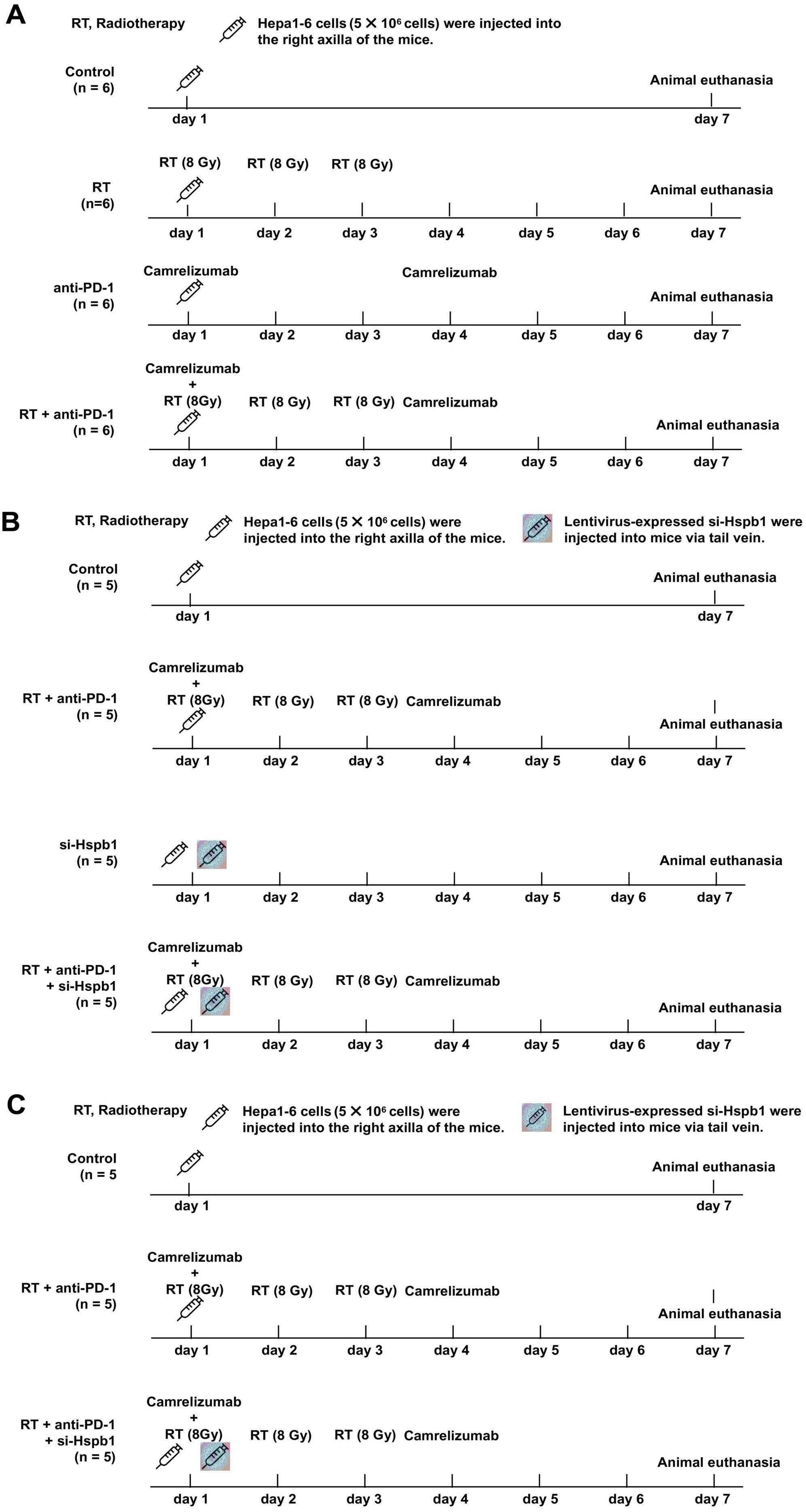
A
RT, Radiotherapy
Hepa1-6 cells (5 X 10⁶ cells) were injected into the right axilla of the mice.
Control (n = 6)
Animal euthanasia
day 1
day 7
RT (n=6)
RT (8 Gy)
RT (8 Gy)
RT (8 Gy)
Animal euthanasia
day 1 day 2 day 3 day 4 day 5 day 6 day 7
anti-PD-1 (n = 6)
Camrelizumab
Camrelizumab
Animal euthanasia
day 1 day 2 day 3 day 4 day 5 day 6 day 7
RT + anti-PD-1 (n = 6)
Camrelizumab + RT (8Gy)
RT (8 Gy)
RT (8 Gy)
Camrelizumab
Animal euthanasia
day 1 day 2 day 3 day 4 day 5 day 6 day 7
B
RT, Radiotherapy
Hepa1-6 cells (5 X 10⁶ cells) were injected into the right axilla of the mice.
Lentivirus-expressed si-Hspb1 were injected into mice via tail vein.
Control (n = 5)
Animal euthanasia
day 1
day 7
RT + anti-PD-1 (n = 5)
Camrelizumab + RT (8Gy)
RT (8 Gy)
RT (8 Gy)
Camrelizumab
Animal euthanasia
day 1 day 2 day 3 day 4 day 5 day 6 day 7
si-Hspb1 (n = 5)
Animal euthanasia
day 1 day 2 day 3 day 4 day 5 day 6 day 7
RT + anti-PD-1 + si-Hspb1 (n = 5)
Camrelizumab + RT (8Gy)
RT (8 Gy)
RT (8 Gy)
Camrelizumab
Animal euthanasia
day 1 day 2 day 3 day 4 day 5 day 6 day 7
C
RT, Radiotherapy
Hepa1-6 cells (5 X 10⁶ cells) were injected into the right axilla of the mice.
Lentivirus-expressed si-Hspb1 were injected into mice via tail vein.
Control (n = 5
Animal euthanasia
day 1
day 7
RT + anti-PD-1 (n = 5)
Camrelizumab + RT (8Gy)
RT (8 Gy)
RT (8 Gy)
Camrelizumab
Animal euthanasia
day 1 day 2 day 3 day 4 day 5 day 6 day 7
RT + anti-PD-1 + si-Hspb1 (n = 5)
Camrelizumab + RT (8Gy)
RT (8 Gy)
RT (8 Gy)
Camrelizumab
Animal euthanasia
day 1 day 2 day 3 day 4 day 5 day 6 day 7

Figure S1 Schedule of the animal experiments. **(A)** Schedule of the animal experiments in control, RT, anti-PD-1 and RT + anti-PD-1 groups. **(B)** Schedule of the animal experiments in control, RT + anti-PD-1, si-Hspb1 and RT + anti-PD-1 + si-Hspb1 groups. **(C)** Schedule of the animal experiments in control, RT + anti-PD-1 and RT + anti-PD-1 + si-Hspb1 groups.

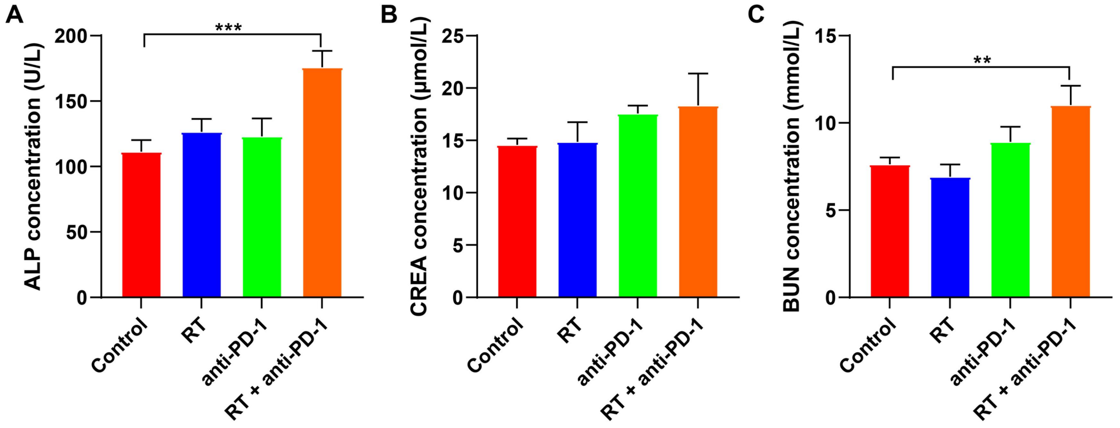

Figure S2 Serum ALP, CREA and BUN levels in mice. **(A)** ALP, **(B)** CREA and **(C)** BUN levels in serum sample of mice were evaluated. **P<0.01, ***P<0.001.

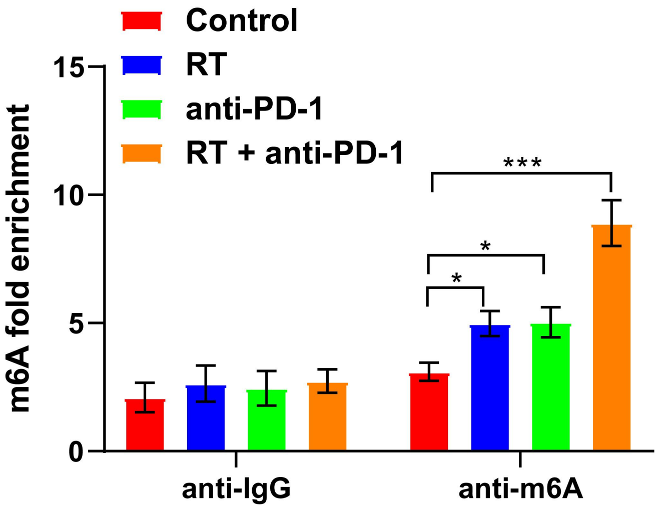

Figure S3 Combination of radiotherapy and immunotherapy impacted the m6A level of Hspb1 in tumor tissues. The m6A level of Hspb1 in tumor tissues among control, RT, anti-PD-1 and RT + anti-PD-1 groups was assessed by meRIP-qPCR. *P<0.05, ***P<0.001.

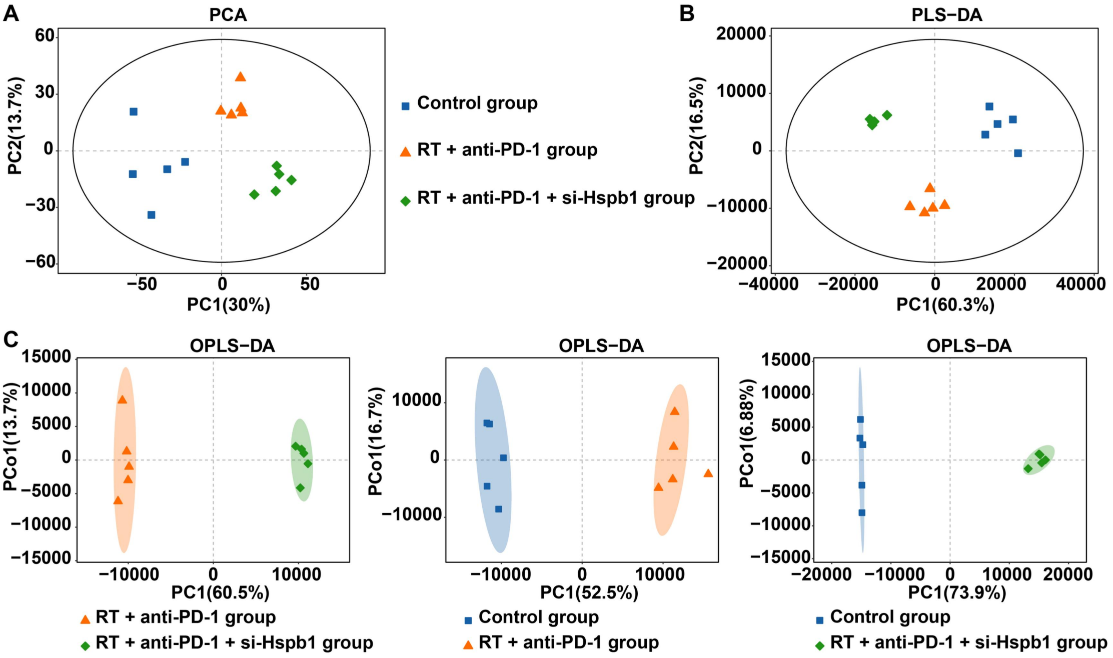

Figure S4 PCA, PLS-DA, OPLS-DA analyses among three groups. **(A)** PCA map. **(B)** PLS-DA map. **(C)** OPLS-DA map.

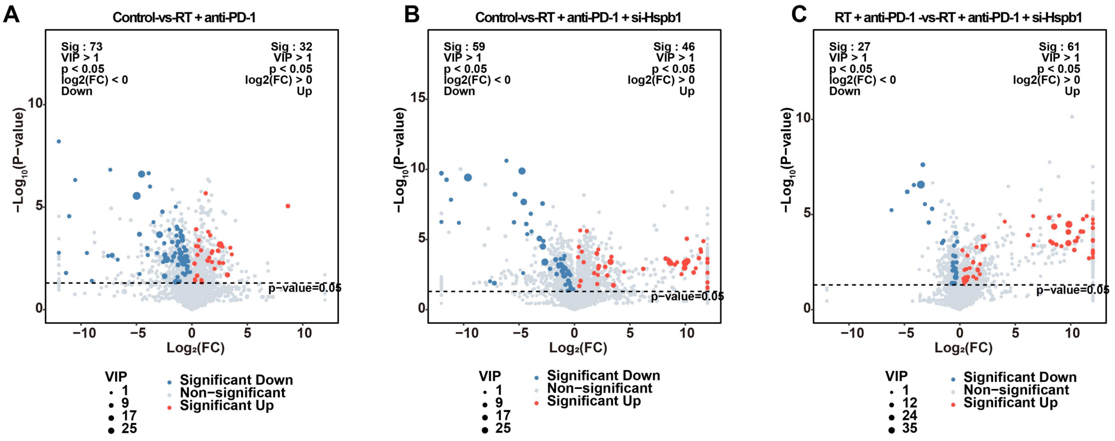

Figure S5 Volcano map of differential metabolites among three groups. **(A)** Differential metabolites between control and RT + anti-PD-1 groups. **(B)** Differential metabolites between control and RT + anti-PD-1 + si-Hspb1 groups. **(C)** Differential metabolites between RT + anti-PD-1 and RT + anti-PD-1 + si-Hspb1 groups.

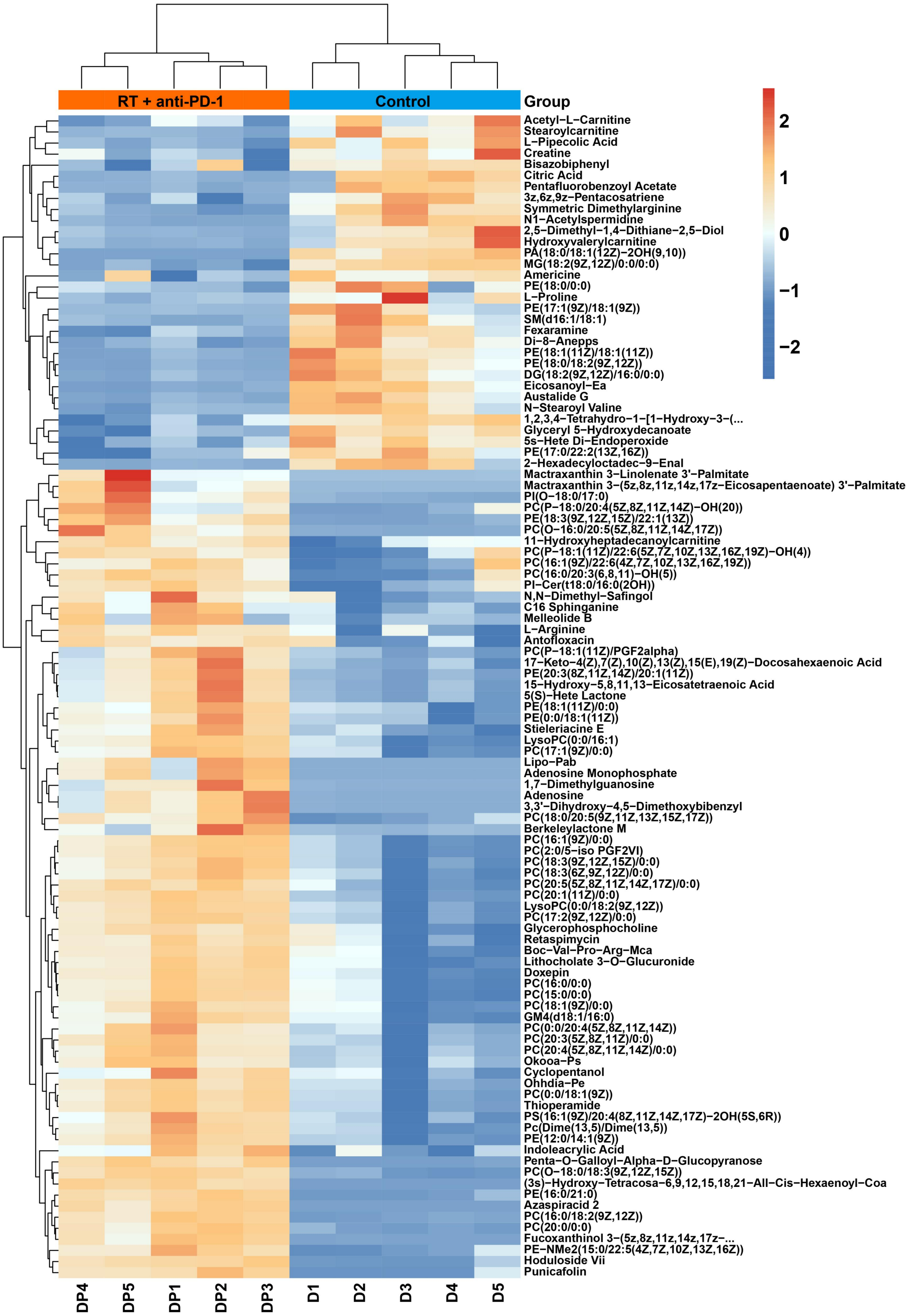

Figure S6 Heatmap showing differential metabolites between control and RT + anti-PD-1 groups.

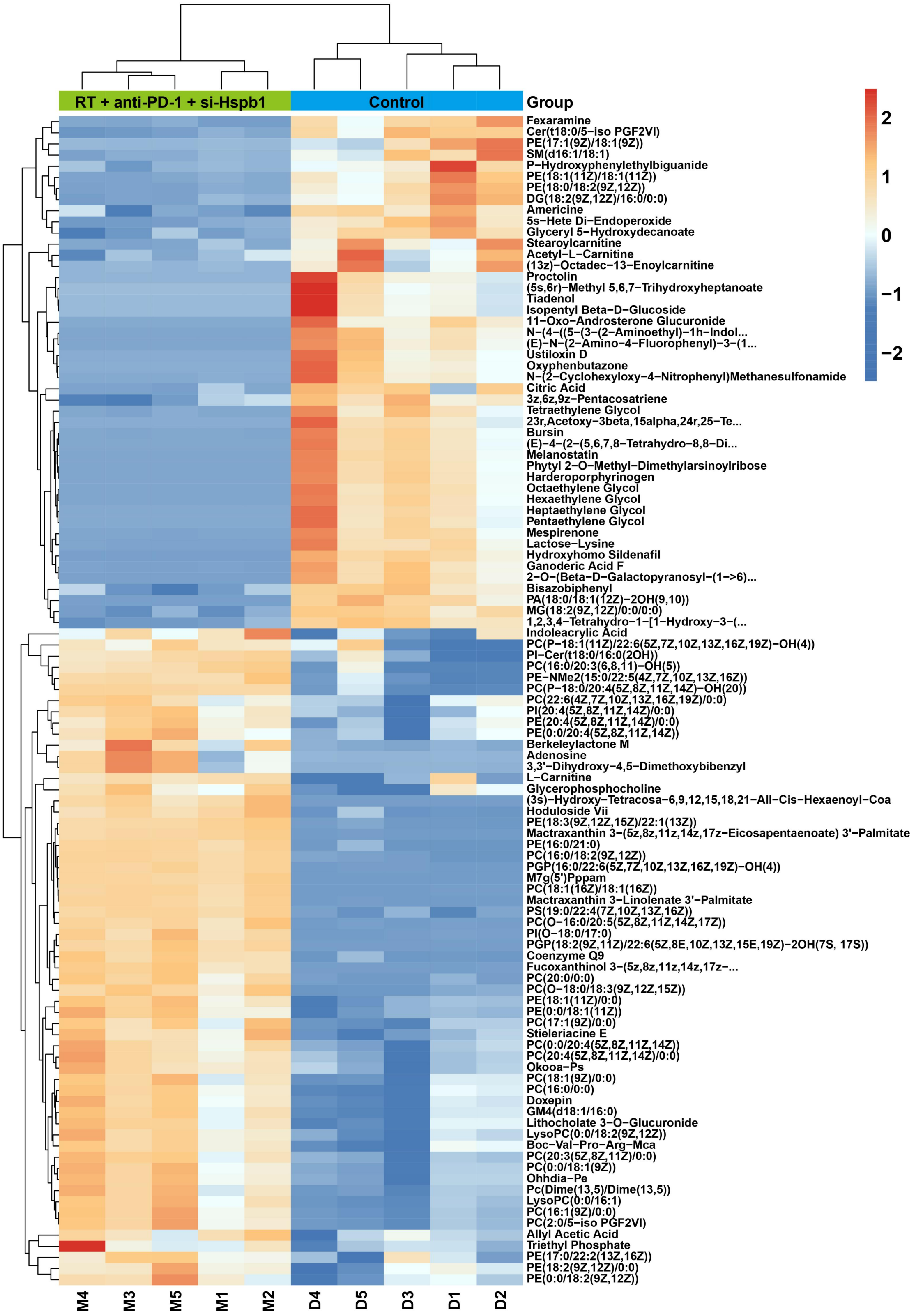

Figure S7 Heatmap showing differential metabolites between control and RT + anti-PD-1 + si-Hspb1 groups.

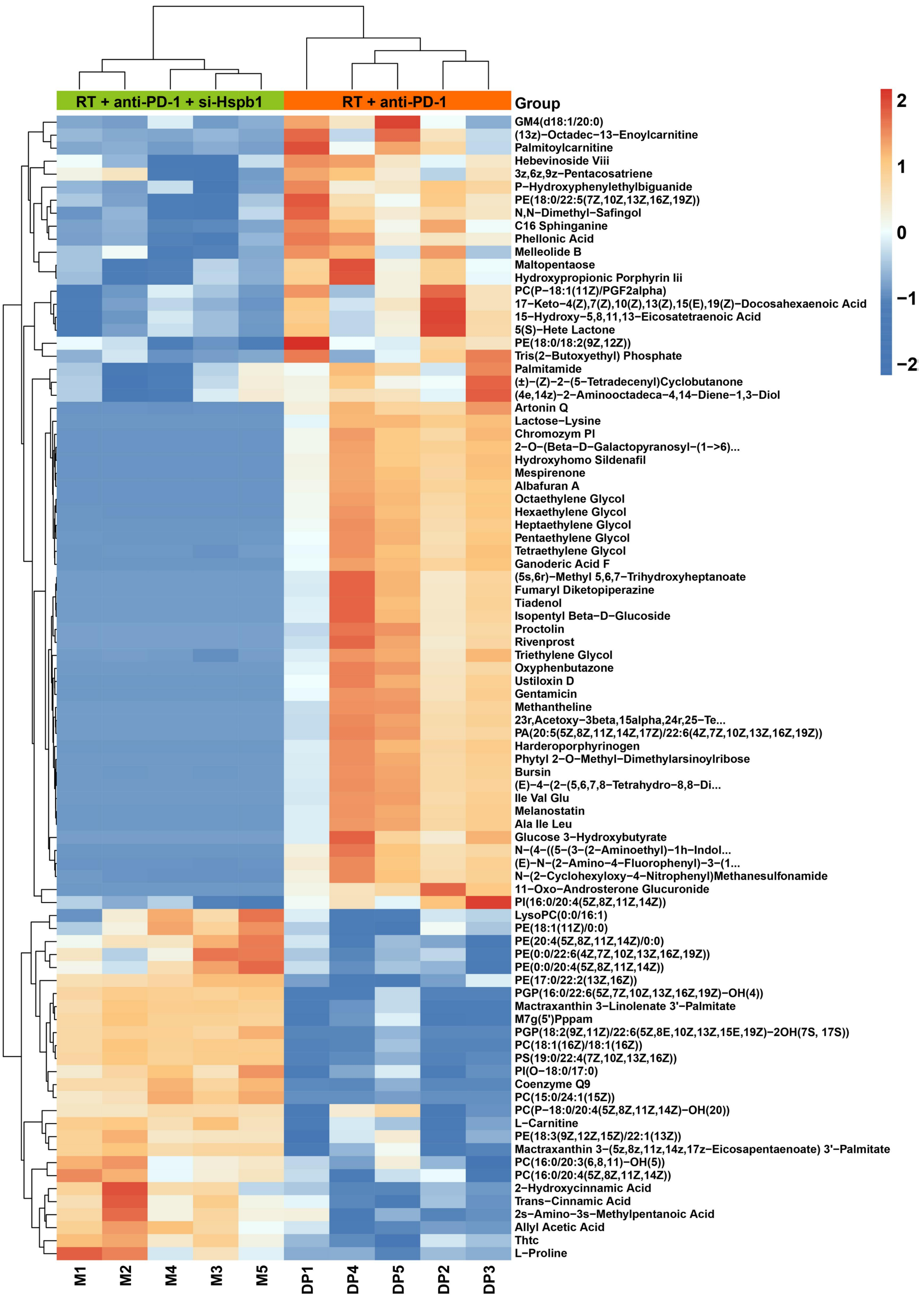

Figure S8 Heatmap showing differential metabolites between RT + anti-PD-1 and RT + anti-PD-1 + si-Hspb1 groups.
